# Motion disrupts dynamic visual search for an orientation change

**DOI:** 10.1186/s41235-021-00312-2

**Published:** 2021-06-26

**Authors:** Emily M. Crowe, Christina J. Howard, Iain D. Gilchrist, Christopher Kent

**Affiliations:** 1 School of Psychological Science, University of Bristol, Bristol, UK; 2 Department of Psychology, Nottingham Trent University, Nottingham, UK; 3 Department of Human Movement Sciences, Vrije Universiteit Amsterdam, Amsterdam, The Netherlands

**Keywords:** Dynamic visual search, Feature change, Motion silencing, Response time, Monitoring

## Abstract

Visual search in dynamic environments, for example lifeguarding or CCTV monitoring, has several fundamentally different properties to standard visual search tasks. The visual environment is constantly moving, a range of items could become *targets* and the task is to search for a certain event. We developed a novel task in which participants were required to search static and moving displays for an orientation change thus capturing components of visual search, multiple object tracking and change detection paradigms. In Experiment 1, we found that the addition of moving distractors slowed participants’ response time to detect an orientation changes in a moving target, showing that the motion of distractors disrupts the rapid detection of orientation changes in a moving target. In Experiment 2 we found that, in displays of both moving and static objects, response time was slower if a moving object underwent a change than if a static object did, thus demonstrating that motion of the target itself also disrupts the detection of an orientation change. Our results could have implications for training in real-world occupations where the task is to search a dynamic environment for a critical event. Moreover, we add to the literature highlighting the need to develop lab-based tasks with high experimental control from any real-world tasks researchers may wish to investigate rather than extrapolating from static visual search tasks to more dynamic environments.

**Supplementary Information:**

The online version contains supplementary material available at 10.1186/s41235-021-00312-2.

## Introduction

Most papers in the visual search literature begin with the description of a daily task which requires us to locate a target object amongst other distracting objects. Rather than studying the daily tasks themselves, psychologists have tended to reduce these examples to specific lab-based visual search tasks in which participants are instructed to search for a pre-specified item amongst competing distractors whilst response time (RT) and accuracy are recorded. Such tasks have the benefit of a high level of experimental control which has resulted in a very rich understanding in this area (e.g. Wolfe & Horowitz, 2017). However, there are some doubts if many of the principles of visual search based on findings from lab-based studies scale-up to more complicated situations (e.g., Kunar & Watson, 2011). One reason for this is because lab-based visual search tasks often fail to capture the full range of classes of real-world searches. Kunar and Watson (2011) conducted a series of experiments in a complex but highly controlled multi-dimensional asynchronous dynamic (MAD) world to assess how basic elements (i.e. motion, luminance changes, high set-sizes, loosely-defined target/template and target uncertainty) of real-world search effected search efficiency. Their overall conclusion was that visual search principles previously shown in the literature do not apply to more complex and ‘realistically’ designed displays. This highlights the need to design lab-based tasks which have high experimental control whilst capturing any specific components of real-world tasks that a researcher may want to understand.

Many real-world visual search tasks encompass more than *just* search. In some dynamic visual search tasks, we must track the changing spatial locations of target and distractor items as they move around the environment. The ability to do this has been extensively studied using the multiple object tracking (MOT) paradigm which requires participants to allocate attention to and continuously track multiple moving objects (see Meyerhoff et al., 2017, for a review). In other real-world tasks, such as CCTV monitoring, the operator must search the monitors and detect the occurrence of any suspicious activity. This task aligns with change detection experiments where people’s ability to detect specific changes (e.g., the suspicious activity) in a visual scene is assessed (see Rensink, 2002, for a review). The real-world tasks researchers seek to understand are complex and often involve components of visual search, MOT and change detection, yet these three paradigms are most commonly discussed and researched in isolation. Clearly, it is advantageous to develop novel tasks that capture and combine components of existing paradigms.

Numerous occupations require search (visual search) in amongst multiple moving objects (MOT) where the goal is to detect a critical event (change detection). For example, lifeguards are required to search dynamic aquatic environments for the occurrence of dangerous events such as drowning; and CCTV operators must monitor a bank of screens to detect suspicious behaviour. In these examples the environment observed constantly changes with high possibilities of occlusion and changing motion patterns: factors that are commonly studied using an MOT paradigm (e.g. Flombaum et al., 2008; Luu & Howe, 2015). In such tasks, the visual environment consists of a set of items where there are numerous potential targets and thus their *status* could change at any point. For example, all individuals in a swimming pool could drown such that, at any point, each could require saving and become a ‘target’. Moreover, these occupations require search for a critical event and thus capture elements of both dynamic visual search and change detection. We therefore developed a novel dynamic visual search for an orientation change task to incorporate these *specific* components of real-world tasks. Importantly, we are using the term *dynamic* to refer to items that are constantly changing spatial location rather than changing feature information (e.g. Van der Burg et al., 2008).

Although the effect of motion on visual search has received a lot of attention in the visual search literature, there remains little consensus on its effect. McLeod et al. (1988) showed that search for targets defined by a conjunction of the features movement and form was done in parallel. They therefore proposed a motion filtering account involving a search system that filtered by movement such that attention could be directed to stimuli with a common movement characteristic (i.e., stationary or moving items), making subsequent search for a remaining single characteristic (e.g. target form) easier. Since then, motion has been shown to aid target detection (e.g., Abrams & Christ, 2005; Franconeri & Simons, 2003), reduce search efficiency (e.g. Kunar & Watson, 2011), or have no effect (e.g. Hulleman, 2009). Such discrepant results emerge due to the different paradigms used to assess the effect of motion on search. Of most relevance to our experiments, Hulleman’s (2009, 2010) work combines an MOT and search paradigm. Participants searched for T’s amongst L’s in either static or moving (i.e. based on MOT) search displays and had similar search slopes for both target present and target absent trials (Hulleman, 2009). In subsequent work, Hulleman (2010) again found no evidence for a difference between static and moving search displays when the task was relatively easy (Experiments 1 and 2) but evidence for a drop in performance when participants were forced to keep track of individual items (i.e., the task was made harder; Experiments 3 and 4). Pratt et al. (2010) also combined an MOT and search paradigm in which participants tracked items moving around a display and had to respond as quickly as possible when they saw the object disappear. In an ‘inanimate’ condition, the items moved in a predictable manner if they collided with each other or the frame and in an ‘animate’ condition an item moved unpredictably without having collided with another item. Response time was faster to targets that underwent animate motion which led the authors to conclude that motion changes that are not due to an external event (e.g., a collision) capture attention. Taken together, this research shows that the effect of motion on search is display- and task- specific which reinforces the need to develop lab-based search tasks that model the components of the real-world task researchers attempt to simulate specifically.

One characteristic of several real-world search tasks that has received little attention in the search literature is that the *status* of an item changes, rendering one item a ‘target’ and the others as ‘distractors’. For example, an individual could be swimming safely one minute and then encounter difficulty shortly after, making this swimmer the target of a lifeguarding search. In low level terms, these types of events are distinguished by changes in motion characteristics or visual appearance and therefore are relevant to the question of the extent to which feature changes in an item can be detected. Some studies have examined the ability to detect such changes within an MOT framework. Sears and Pylyshyn (2000) showed that target form changes were identified faster than non-target form changes and Bahrami (2003) showed participants were more likely to detect color and shape changes in targets than distractors. Vater et al. (2016) showed that changes in target motion (a change in speed) were detected faster than changes in target form (a change in shape). In these studies, however, the target item was known to participants prior to the onset of a trial which is not representative of many dynamic search tasks in which all items in a display could potentially become a target.

Pylyshyn et al. (2008) used a probe detection task where participants were required to monitor for the occurrence of small dots that could occur anywhere on the screen. Participants completed a standard tracking condition in which they had to both track the targets and detect the presence of a probe and a control condition where they were not required to track targets. In both conditions, participants detected more probes on static non-target items than moving non-target items suggesting that the motion of non-target items impaired detection of the probe. To better understand the extent to which motion impairs the detection of a probe, collecting RT is beneficial as typically done in the visual search literature but less commonly used within an MOT framework. In other related work, Tripathy and Barrett (2004) developed a task which assessed participants’ ability to detect a deviation from the linear trajectory of moving items. In their Experiments 3 and 4, all items were potential targets (i.e., could deviate from a linear trajectory) thus requiring participants to monitor the trajectories of all items simultaneously. They showed that when one item changed trajectory (i.e., became the target), the detection threshold to identify this change rose steeply with the number of items within a display. However, few other studies have investigated the situation where there are numerous potential targets, and thus must be monitored, and target identity is only apparent later. More research is required to better understand how people track objects while searching for a target that is signalled by a change in status and other *types* of changes, such as feature changes, also require consideration.

Here, we sought to investigate the effect of motion on the detection of a visual change within a dynamic visual search framework. In two experiments, we introduce a novel dynamic visual search task for a change event. Experiment 1 explored the effect of set size and object motion (stationary or moving) on change detection time and Experiment 2 explored whether there was an additional cost associated with detecting a feature change that occurred on a moving target compared with a static target.

### Experiment 1

Experiment 1 examined the effect of set size and object motion on the time to detect an orientation change in a Gabor patch. This study was pre-registered on the Open Science Framework (OSF, https://osf.io/6gs72/).

#### Participants

Thirty undergraduate students from the University of Bristol (19 female, with a mean age of 19.87 years, SD = 2.01) took part in return for course credit. Participants in both experiments had self-reported normal or corrected-to-normal vision.

#### Design

A repeated measures design with set size (1, 2, 3, 4, 5, 6, 7, or 8 targets) and object motion (static or moving) as the independent variables and time to detect an orientation change as the dependent variable was used.

#### Procedure

**Fig. 1:**
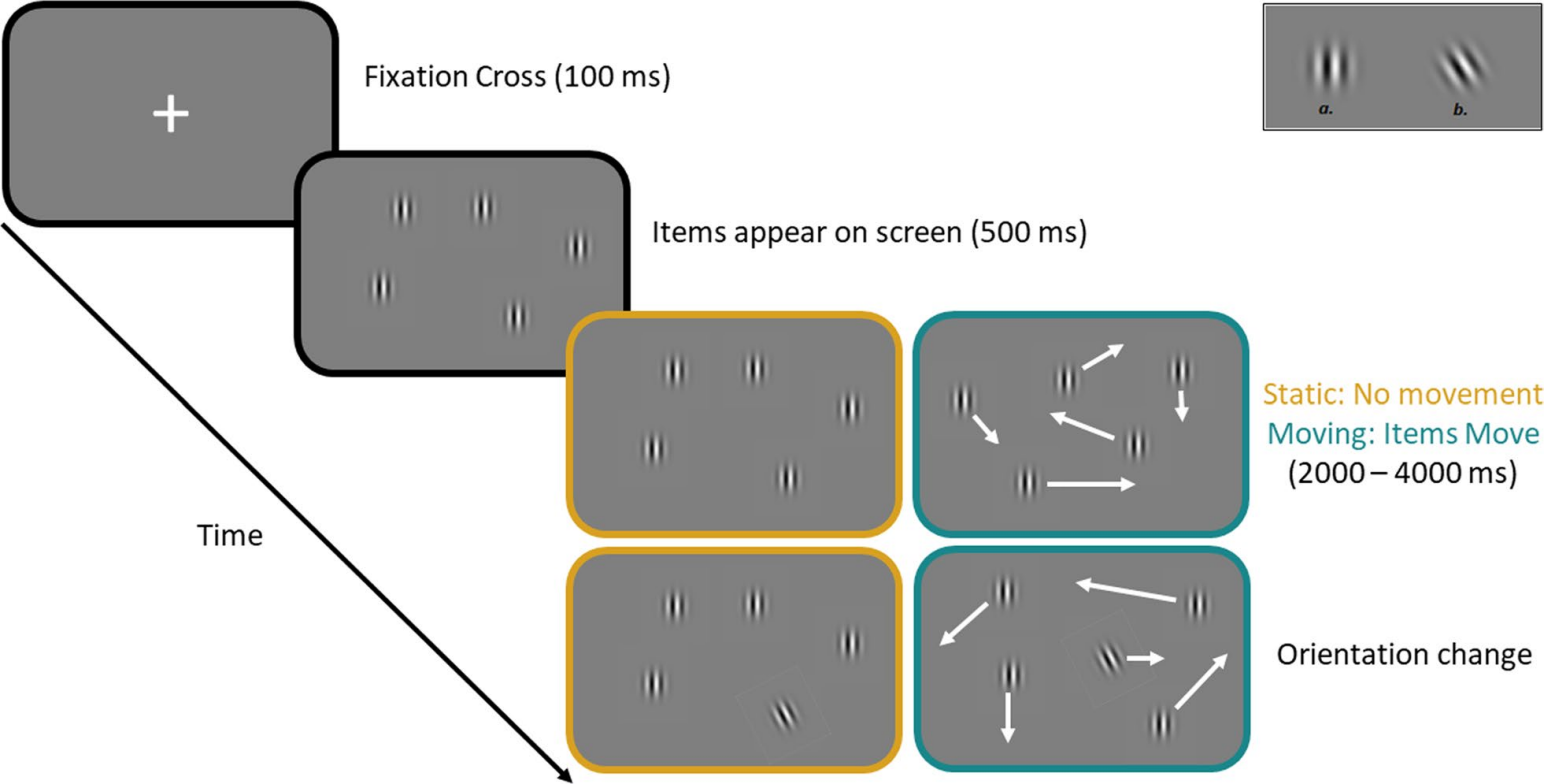
Timeline of the task. Each trial beings with a fixation cross. The items then appear on screen for 500 ms. In the static condition (golden screen), the items do not move. In the moving condition (turquoise screen), the items move around the screen. After a random interval between 2,000 and 4,000 ms, one item will undergo an orientation change. The panel in the top right shows the starting orientation of all items (**a**) and the rotated orientation of the target item (**b**)

Participants sat approximately 40 cm away from a 21″ LCD monitors with a resolution of 1920 * 1080 pixels refreshing at 60 Hz used to present stimuli. Participants were tested in groups in a large computing laboratory (which precluded completely standardising luminance and viewing distance, so we report RGB and pixel values). Stimuli consisted of Gabor patches (striped sinusoidal gratings within a Gaussian envelope, and mean RGB value of 128, 128, 128, matching the background color, with maximum and minimum RGB values of 255, 255, 255 and 0, 0, 0 representing 100% contrast). The visible diameter of the Gabor was 64 pixels. The background remained a uniform grey (RGB 128, 128, 128) throughout the experiment. At the beginning of each trial, a white fixation cross (“+”) was displayed in the centre of the screen. A number of targets (between 1 and 8) were then displayed on screen in random locations (at least 70 pixels away from the screen edge and other targets). At the start of the trial, all items were oriented vertically. In the stationary condition, the targets remained in their original locations for the entirety of the trial. In the motion condition, the targets began moving after 500 ms and targets moved along randomly selected trajectories at a constant randomly chosen speed between 85 and 254 and pixels per second. If targets collided with the screen edge they rebounded. If targets collided with one another they rebounded off each other (i.e., ballistic motion). After a random duration between 2000 and 4000 ms had elapsed, one randomly selected target would change orientation by a 30° rotation anti-clockwise (see Fig. 1, top right corner). One item underwent an orientation change of every trial such that there were no target-absent trials. Participants were instructed to press the left mouse button of a standard USB mouse as soon as they detected a change. After a response was recorded, a blank screen was displayed for 1000 ms before the next trial commenced. There were two blocks of 240 experimental trials (i.e. 30 trials per condition), with object motion and set size randomly intermixed across blocks. There were five practice trials.

## Results and discussion

**Fig. 2:**
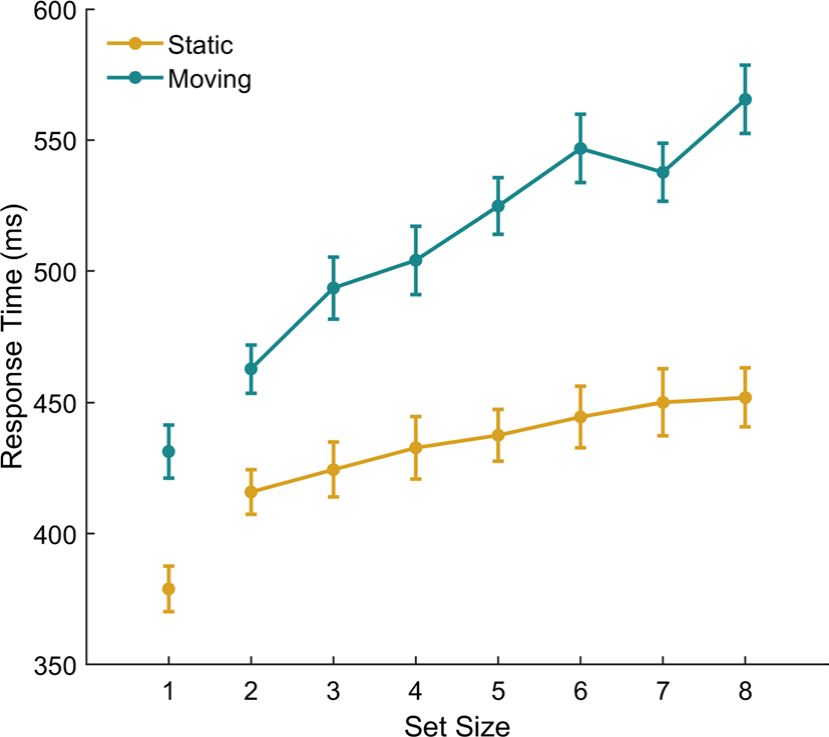
Mean RT for each set size and display type. Error bars show standard error

All data (from both experiments) are available from the University of Bristol data repository (https://doi.org/10.5523/bris.1ayzsmttl78pg2wymtkevg2zld). Response times smaller than 200 ms (< 1%) or greater than 4000 ms (1%) were removed and not analysed further under the assumption that these responses reflected anticipations and attentional lapses, respectively. Since we did not include target-absent trials, we inspected the individual level data to identify any participants who did not engage with the task properly. Specifically, we checked for any evidence for a second ‘guessing’ peak which would suggest that a participant applied a time threshold strategy and just responded after a set period of time without actually detecting an orientation change. Based on this analysis, the data from two participants was removed because their data suggested they either produced too many anticipatory responses or were inattentive (a summary of this analysis can be found in the Supplementary Information, Figures S1–S5). For each participant, we calculated the median RT in each condition. We calculated the median RT because the distributions for individual participants were positively skewed. Figure 2 shows the mean RT across participants for each set size and object motion condition. For the analysis, we only included set sizes two to seven because we consider trials with a set size of one to be a qualitatively different task that does not constitute search. We refer to all objects prior to the change event as items. Following the orientation change, we refer to the item that underwent the orientation change as the target and the items that did not change their orientation as distractors. There was an effect of set size, with RT being slower for larger set sizes, *F*(6,162) = 23.24, *p* < 0.001, *η*_p_^2^ = 0.463, and responses were faster to stationary (*M* = 439 ms; SD = 58 ms) compared with moving (*M* = 519 ms; SD = 69 ms) search displays, *F*(1,27) = 97.52, *p* < 0.001, *η*_p_^2^ = 0.783. There was also an interaction, *F*(6, 162) = 5.47, *p* < 0.001, *η*_p_^2^ = 0.138, with a greater effect of motion at larger set sizes.

Responses were faster for the stationary compared with moving condition. As supported by the interaction, the slope is flatter for the stationary displays, indicating more efficient search in the stationary than the moving displays. We included the set size of 1 to assess whether there was any evidence for an effect of motion when only one item was present in the display although this condition is not a visual search task as such because the participant knows which item will become the target and there are no distractors. Even when all the participant’s attention could be allocated to that single item, RT is slower when that item is moving thus suggesting motion disrupts the detection of the orientation change, even for a single item. However, further experiments are required to fully understand the effect of motion within this task. Experiment 2 therefore introduced displays consisting of both stationary and moving items and examined the effect of either a moving or static item undergoing the orientation change. In this way we were able to manipulate the presence or absence of motion in the target item to measure this specific effect of motion on performance.

### Experiment 2

Experiment 2 investigated whether motion of the target slowed detection of the orientation change to gain insight into whether target motion itself disrupts feature change detection. This study was pre-registered on the OSF (https://osf.io/9t3kg/).

#### Participants

Thirty-one participants^1^ (26 females, with a mean age of 16.70 years, SD = 0.82) volunteered to participate as part of an outreach programme at the University of Bristol and provided written informed consent.

#### Design

A repeated measures design with set size (2, 4, 8) and object motion (all stationary (henceforth ‘stationary’), all moving (henceforth ‘moving’), mixed display with static target (henceforth ‘mixed display -static target’), mixed display with moving target (henceforth ‘mixed display -moving target’)) as the independent variables and time to detect an orientation change as the dependent variable was used.

#### Procedure

The procedure was identical to Experiment 1, with the following exceptions. In both the mixed display—static target or mixed display—moving target conditions, exactly half the stimuli moved, and half remained static. Moving items rebounded off static items, each other, and the screen edge. One moving (in the mixed display—moving target) item or one static (mixed display—static target) item changed orientation between 2000 and 8000 ms after the start of the trial. All conditions were randomised in 10 blocks of 36 trials.

## Results and discussion

**Fig. 3:**
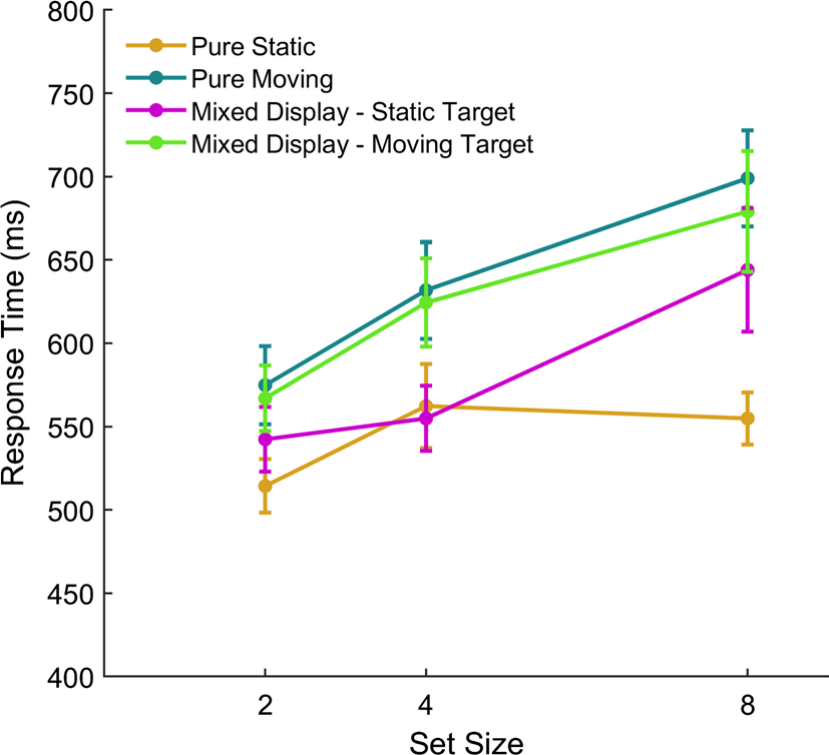
Mean RT for each set size and display type. Error bars show standard error

We conducted the same initial screening of the raw data to identify any participants who displayed behaviour consistent with a guessing strategy. Two participants’ data suggests they either produced too many anticipatory responses or were inattentive (a summary of this analysis can be found in the Supplementary Information, Figures S6–S9). In line with Experiment 1, response times shorter than 200 ms (< 3%) or greater than 4000 ms (1%) were removed and are not analysed further. For each participant, we calculated the median RT in each condition. Figure 3 shows the mean of these median RTs for each set size and display type. There was an effect of display type: RT was fastest in the stationary condition and slowest in the moving condition, *F*(3,84) = 25.53, *p* < 0.001, *η*_p_^2^ = 0.477. There was also an effect of set size, with RT increasing as set size increased, *F*(2,56) = 20.37, *p* < 0.001, *η*_p_^2^ = 0.421. An interaction was also observed, *F*(6, 168) = 3.21, *p* = 0.005, *η*_p_^2^ = 0.103, with the effect of speed being greater at larger set sizes. These results replicate our findings from Experiment 1: RT is slower in pure moving compared with pure static displays and this difference is greater at larger set sizes. Bonferroni corrected pairwise comparisons showed that in mixed displays consisting of both static and moving objects, RT was faster when the orientation occurred on a static item (*M* = 580 ms; SD = 154 ms) than a moving item (*M* = 623 ms; SD = 173 ms), *p* = 0.011. This suggests that motion possessed by the target itself disrupted the detection of the orientation change. RT was slower in pure static displays (*M* = 544 ms; SD = 109 ms) compared to mixed displays where the target item was static (*p* = 0.003). This shows that distractor motion in the mixed displays slows the detection of a stationary target indicating a detrimental effect of distractor motion on top of that which can be attributed to the target itself. There was, however, no evidence for a difference between the pure moving (*M* = 635 ms; SD = 158 ms) and mixed with a moving target (*p* = 1). Although a somewhat speculative interpretation, this could suggest that the presence of some motion in the display is sufficient to disrupt detection and that this effect saturates such that more motion does not further disrupt detection.

## General discussion

We introduce a novel dynamic visual search for a change event task combining elements of standard MOT, visual search and change detection paradigms. Using this task, we presented two experiments that show that motion possessed by both distractor and target items independently slow the detection of an orientation change in a moving Gabor. Search is relatively robust to effects of motion when search is easy but motion can slow search when the task is harder (Hulleman, 2009). Since it is difficult to determine the difficulty of our task relative to those used in previous work and we did not assess the effect of increasing the difficulty (e.g., by reducing the contrast of the Gabors), we do not consider it advantageous to directly compare our findings to other research investigating the effect of motion given the large differences in the stimuli used. We will therefore focus on possible explanations for our finding that motion slows detection in dynamic visual search for an orientation change.

Previous research has shown that motion silences detection of feature changes. In a series of experiments, Suchow and Alvarez (2011) showed that objects changing in hue, luminance, size and shape appear to change less rapidly when they move therefore highlighting a motion-induced failure to detect change. Suchow and Alvarez (2011) attribute the silencing effect to motion on the retina rather than motion in space. Faster moving items spend less time at any location on the retina and this brief exposure may not be sufficient to detect feature changes. In our *moving* conditions, the items always move but we did not track participants eye movements so we do not know how participants moved their eyes. It is possible that participants continually saccade from target to target, exposing them all to brief periods of high visual resolution (Landry et al., 2001). Alternatively, participants may focus at the centroid of targets during tracking (Fehd & Seiffert, 2008; Yantis, 1992) or even maintain fixation around the centre of the screen. Irrespective of the eye-movements used, this task would have resulted in motion in both space and on the retina and, therefore, these results would fit with Suchow and Alvarez’s motion silencing account for feature changes. We consider this to be contributing to the effects reported here alongside lower-level interference from motion incurred from the luminance transients produced by motion.

In our Experiment 1 here, the search slope from 2 to 8 items was 9.7 ms per item for the static display which is below the 10 ms/item typically thought to represent ‘pop-out’ in a display (Theeuwes, 1995a; Trick & Enns, 1997). This suggests that in our static display, the transient signal pops-out whereas in the moving displays, the transient signal is somewhat masked by the motion. In line with the idea of motion silencing, it could be the motion itself that masks the orientation change or, alternatively, it could be that other transients also contribute to masking this signal. In our moving conditions, objects rebounded off the boundary of the experiment and each other after a collision thus generating transient events which may have also contributed to masking the signal. In support of the view that collisions may attract attention away from other events, Landry et al. (2001) showed that participants made more saccades to targets of potential collisions, Fehd and Seiffert (2010) suggested gaze might shift from a centroid-looking strategy to a target when task items were in close proximity to each other, and Vater et al. (2017) showed that target collisions attracted gaze in the direction of such collisions in an MOT task. It therefore seems possible that the higher occurrence of additional transients in our moving condition (Experiment 1) might attract attention and slow participants' ability to detect the task-relevant transient, namely the orientation change. In our Experiment 2 here, the frequency of transient collision events is the same in both of the mixed displays. Therefore, these collisions will likely be distracting in both of these conditions. Slower detection seen when it is a moving item that undergoes the orientation change suggests that motion possessed by the target additionally slows detection, likely due to lower level masking by luminance transients as the target translates around the display.

There are three strategies that participants could have used to complete this task. One possibility is that participants monitored for the change event (the transient signal) or, alternatively, they could have searched for the target using the template of the oriented Gabor. Another possible but unlikely strategy is that participants engaged in multiple identity tracking (MIT; Oksama & Hyona, 2008) whereby they assigned each target an identity and continuously updated the identity-location bindings of each item. Given the attentional load and difficulty associated with this latter strategy it is unlikely that participants engaged in MIT consistently, especially at larger set sizes. Irrespective of the strategy used, which could differ both within and between participants, our results show that motion of the distractor and target items slows the detection of the orientation change event. Future research might investigate the impact of various strategies for search in dynamic scenes because this would have clear practical implications in terms of training and effective search for feature changes among dynamic scenes.

As discussed above, it is possible that participants used the target template of an oriented Gabor to guide their search. Since previous research has shown that search is more efficient for very specific target templates (e.g., Malcolm & Henderson, 2009, 2010; Vickery et al., 2005; Wolfe et al., 2004), using this strategy would have likely aided performance here. Future research might investigate the extent to which our results generalise to search tasks where the target is not well specified which is more reflective of the real-world. In lifeguarding, for example, active and passive drowning consist of very different features which highlights one way in which the ‘target template’ is poorly defined (Laxton & Crundall, 2018). Research has shown that, when presented in the same context, the target template is often biased towards information that facilitates search performance. For example, Navalpakkam and Itti (2007) showed that participants used a target template for a line oriented at 60° when searching for a target oriented at 55° among those oriented at 50° and Becker (2010) showed that participants used a target template of red when searching for an orange target amongst yellow distractors. A less specific template limits the efficacy of using such biases in one’s template and thus highlights the increased complexity in real-world searches for poorly defined targets. Bravo and Farid (2016) have shown that participants can learn multiple target templates for a single target and that they can voluntarily switch among these which highlights the possible benefits of training target templates and should be considered in search occupations.

A limited number of studies (cf. Tripathy & Barrett, 2004) have used search paradigms in which each item is a potential target at the start of the trial. In such studies, there are no distractors in the sense of being items that the participant could actively ignore or inhibit (i.e., ‘traditional distractors’) until the point at which one item underwent a change and became the target. Future research should explore the effect of *actual* distractors or other salient sources of distraction in the visual environment on performance in dynamic search tasks. In a lifeguarding situation, for example, it remains to be seen whether motion of the waves in a wave pool would be detrimental to the detection of a drowning incident, in addition to the motion of the swimmers (potential targets) themselves. In MOT, participants can strategically split their attention unequally (Crowe et al., 2019) and, in visual search, task relevance predicts the gaze of participants monitoring an array of CCTV screens (Howard et al., 2011). Therefore, it is likely that certain locations (e.g. a wave pool) and targets (e.g. a younger swimmer who is at a greater risk of danger) might be searched with greater priority than others in real world analogues of our paradigm.

We developed our task to capture important components of real-world searches that could be studied in a controlled experimental setting. Although our task is still largely artificial, our findings have implications for the occupations that contributed to the development of this task such as lifeguarding and CCTV monitoring. Our main finding is that motion is detrimental to search performance (efficiency) and, therefore, training with these types of scenes should be emphasised. Since expert CCTV operators look at task relevant areas earlier than non-experts (Howard et al., 2013), there is promise that training may facilitate performance. A consideration for current practices in CCTV monitoring, for example, is to limit the number of screens being monitored by each operator. Since adding more potential targets (i.e., more screens) makes observers less likely to detect an event, imposing limits on the number of screens each operator is required to monitor could reduce the number of critical events that are missed.

## Conclusions

We developed a task combining MOT, visual search, and change detection in an attempt to better capture components of complex real-world searches. We find that that motion negatively affects event detection in a dynamic visual search context. In line with accounts of motion silencing (Suchow & Alvarez, 2011), motion possessed by the target item itself and in surrounding items are two independent sources of disruption to the detection of the change event. These results have important implications for occupations in which search for the detection of a change event is required.

## Supplementary Information

**Additional file 1.** Supplementary materials including additional data analyses.

## Data Availability

Data are available at the University of Bristol data repository, data.bris, at https://doi.org/10.5523/bris.1ayzsmttl78pg2wymtkevg2zld

## Abbreviations

MOT: Multiple object tracking
RT: Response time
MIT: Multiple identity tracking
OSF: Open science framework

## Footnotes

1 The data was collected as part of an outreach programme which resulted in over-recruitment relative to our planned sample size (N = 16). Since all data was collected on the same day, at the same time, we chose to analyse all the data.
