# Supplementary material for: Motion disrupts dynamic visual search for an orientation change
Source: Cogn Res Princ Implic. 2021 Jun 26;6:47. doi: 10.1186/s41235-021-00312-2 (PMC8236006; doi:10.1186/s41235-021-00312-2)
Supplement: Supplementary file 1 — Additional file 1. Supplementary materials including additional data analyses. [file 41235_2021_312_MOESM1_ESM.docx]

# Supplementary Information

There were no ‘target-absent’ trials included in the experiment and, therefore, response time (RT) is a metric of how long it takes a participant to detect the change (there was no localisation of the change). Although we did not include target-absent trials (and so it is possible that participants guessed that there had been an orientation change rather than actually detecting it on some trials), the analysis presented below clearly indicates that the majority of participants on the vast majority of trials were completing the task as intended, namely only responding once they actually detected an orientation change.

There are several ways in which participants could have completed the task *incorrectly* (i.e., not in line with the task instructions which were to press the mouse button when they detected the orientation change).

First, it is possible that participants just continuously pressed the mouse button to finish the experiment as quickly as possible. Since there are very few responses faster than 200 ms in both experiments (< 1% in Experiment 1; see Figure S1 and <3 % in Experiment S2; see Figure S6), this strategy is unlikely to have been used on many trials. We remove all responses shorter than 200 ms such that the majority of these ‘anticipatory’ responses (or ‘guesses’) were removed from the analysis. This approach is supported by our analysis below.

A second possibility is that participants attempted to complete the task to some extent but applied a certain time threshold after which they assumed the orientation change had occurred such that they gave up on that trial and responded rather than actually detecting the orientation change. If a participant did guess according to such a time threshold, we would expect this to be reflected in the RT-distribution for each individual participant. Specifically, we would expect the RT distribution to contain two peaks whereby the first peak represents the responses to an orientation change (i.e., in line with task instructions) and the second peak represents guesses centred around a given participants’ time threshold (i.e., given participants will have some variability in their perceived lapse of time).

The trials completed correctly by participants and any trials in which they might have applied a time threshold will mix in the RT-distributions. We therefore time-lock RTs relative to both the start of the trial and the orientation change. When we time-lock to the start of the trial participants' 'real' responses will be smeared out due to the variability in the time from the trial start to the orientation change. When we time-lock to the orientation change the 'guess' responses will be smeared out because participants' time threshold would be applied relative to the start of the trial (i.e., if they don't detect the orientation change, they cannot apply a time threshold relative to that event).

Figure S1 shows the RT-distribution for all participants combined, Figure S2 shows the RT-distribution from the onset of a trial, and Figure S3 shows the RT-distribution from the orientation change for each participant in Experiment 1. Overall, these figures do not show evidence for a second peak that would be consistent with the time threshold strategy. The distributions are typical for response time tasks of this nature (unimodal with a positive skew, e.g. ex-Gaussian). However, since it could be argued that there is evidence for a second peak in some participants data (e.g. Participants 8 and 15), we further analysed these data.


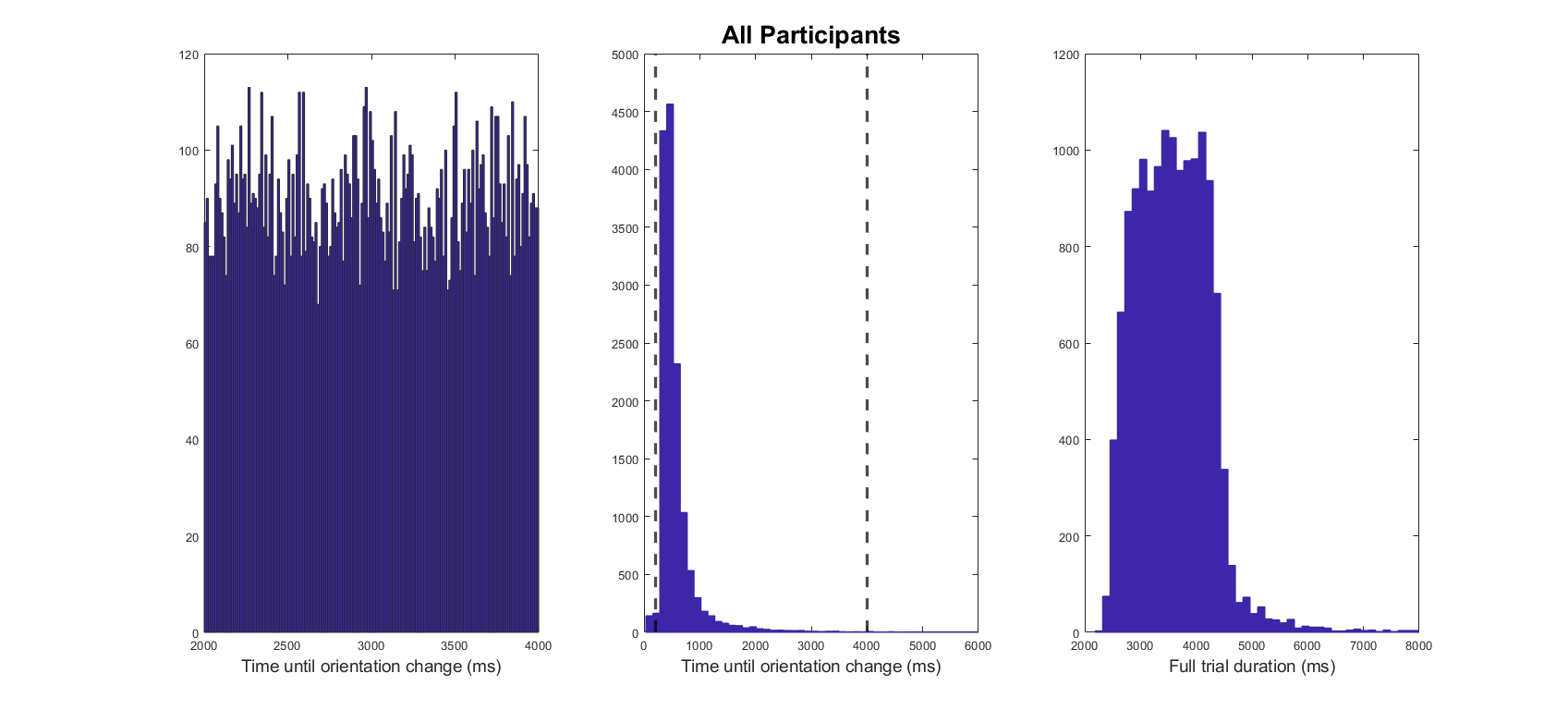
*Figure S1.* Response time distributions for all participants in Experiment 1. Vertical dotted lines (middle panel) show the cut-offs used in our experiment (200 ms – 4000 ms).

*
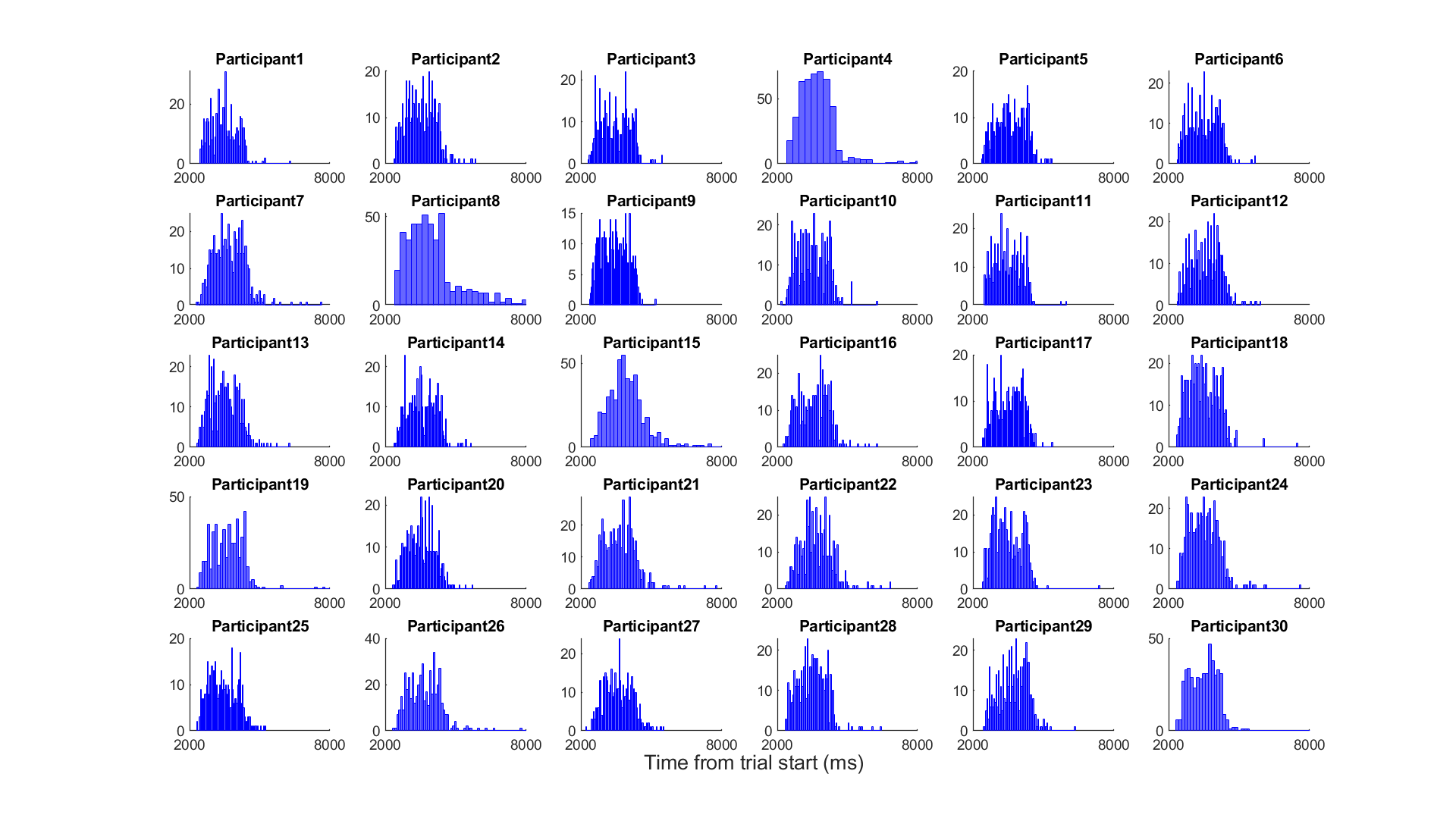
Figure S2.* Response time distributions from trial start to participant response for each individual participant in Experiment 1.

*
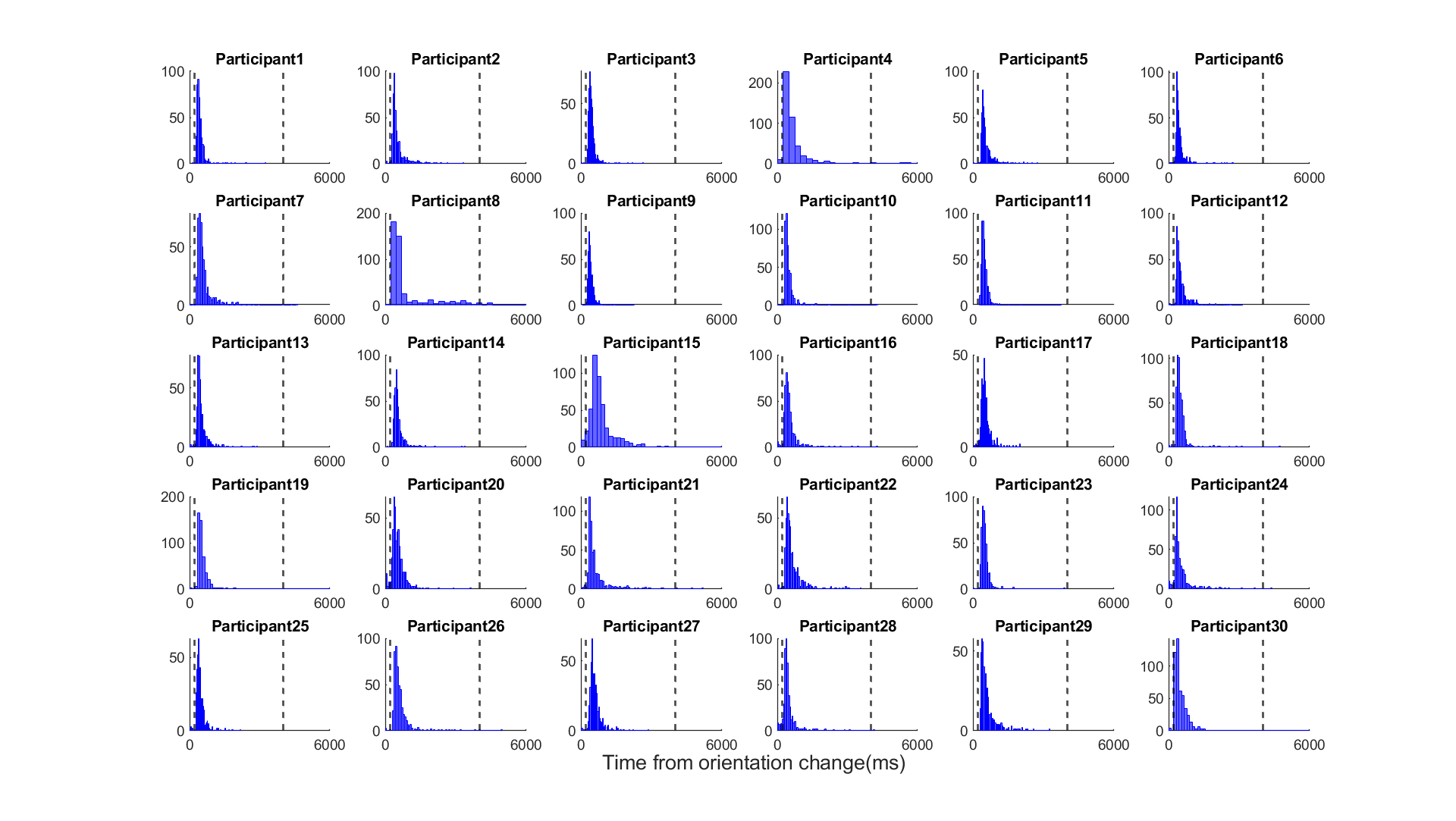
Figure S3.* Response time distributions from orientation change to participant response for each individual participant in Experiment 1. Vertical dotted lines represent the cut-offs used in our experiment (200 ms – 4000 ms).

To gain more insight into the RT distributions for each individual participant, we took the reciprocal of the response time for each trial to obtain the speed. Assuming the RT distributions are approximately ex-Gaussian, this should make the speed-distributions more normal. We then vincentized the data. The rank-ordered speeds for each participant were divided into 10 bins and the mean speed was then calculated for each bin. Figure S4 shows examples for two participants. The left panel shows a participant who we think completed the task according to the instructions and did not show signs of guessing. The right panel shows a participant who does display some evidence for guessing.

**Participant 30**

**Participant 15**


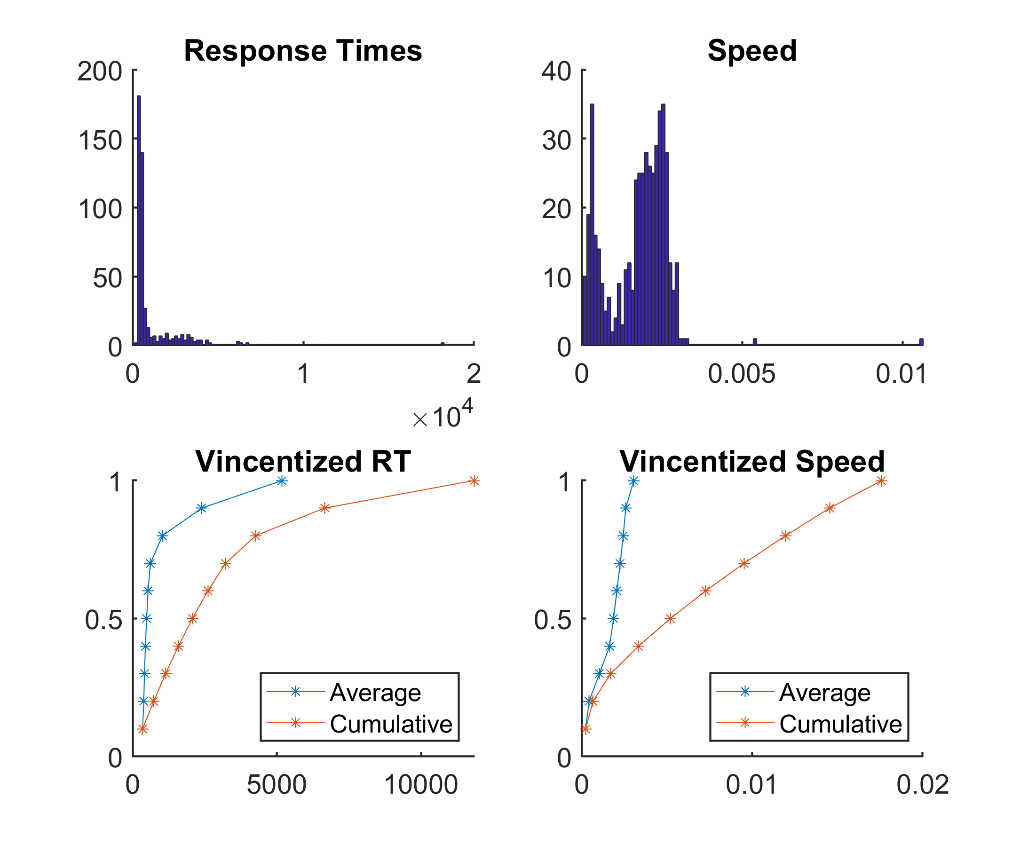

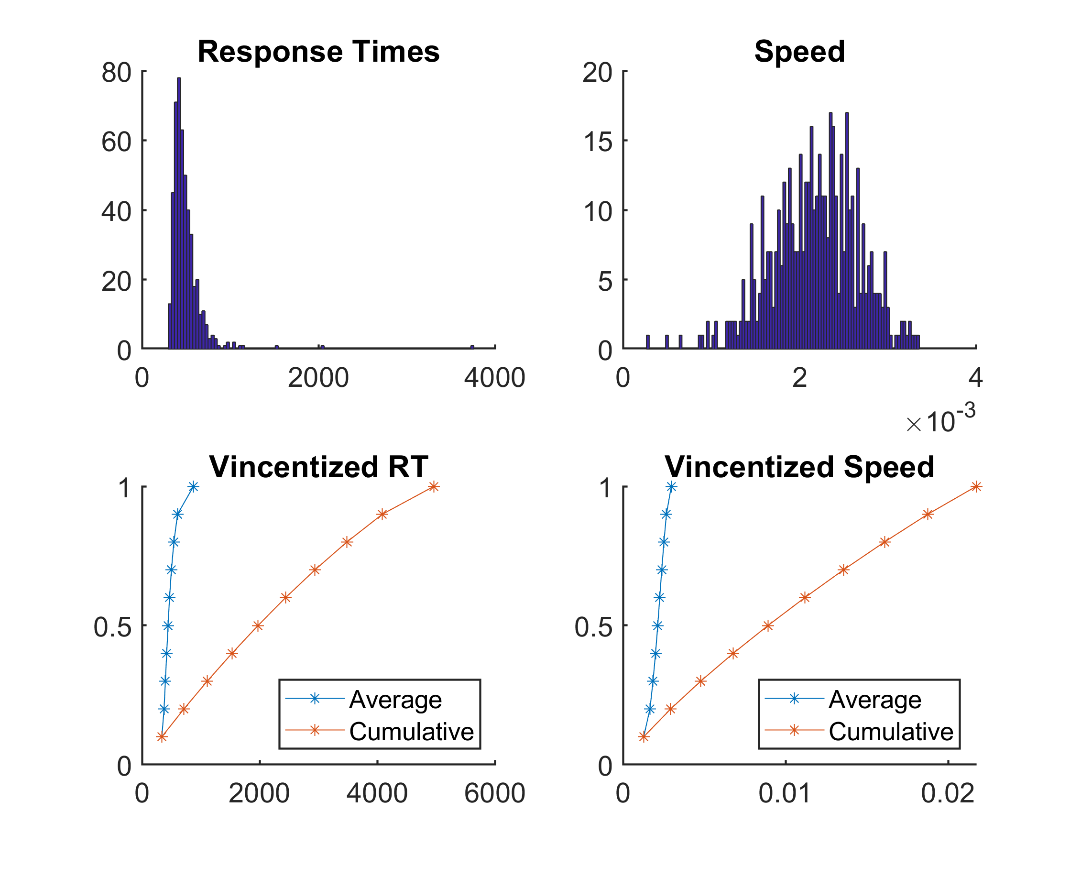


*Figure S4.* Examples of the processing steps for two participants. Data is RT from orientation change.


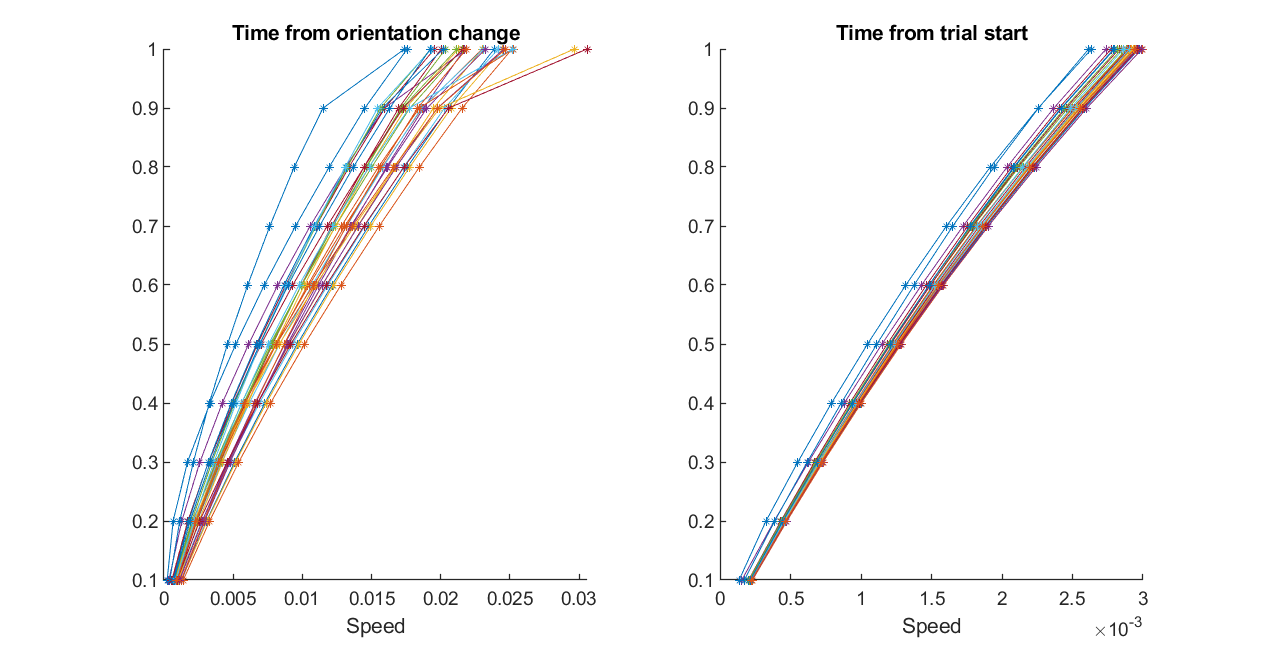
Figure S5 shows the cumulative frequency of the vincentized data for each individual participant. A linear slope can be seen for the majority of participants which is consistent with a normally distributed speed distribution and, in turn, an ex-Gaussian RT distribution. This strongly suggests that participants were responding to the stimulus event and not deploying any other strategy. Four participants deviate from this to some extent (including the participant whose data is shown in the right panel, Figure S4). Following this detailed analysis, we have removed participants 8 and 15 from the analysis reported in the manuscript. We do not believe the other participants display behaviour consistent with guessing throughout the whole experiment and thus keep them in the analysis. Of course, we cannot rule out the possibility that they guessed on a limited number of trials, but we are convinced these are the minority of trials and are likely to be relatively evenly distributed across the conditions. Given the skew in the individual participants data, we use individual participants medians in the analysis reported in the manuscript. Importantly, we note that using means or medians make no qualitative difference to the findings or the conclusions of the manuscript.

*Figure S5.* Vincentized data for each participant in Experiment 1. Left panel shows RT from orientation change; right panel shows RT from trial start.


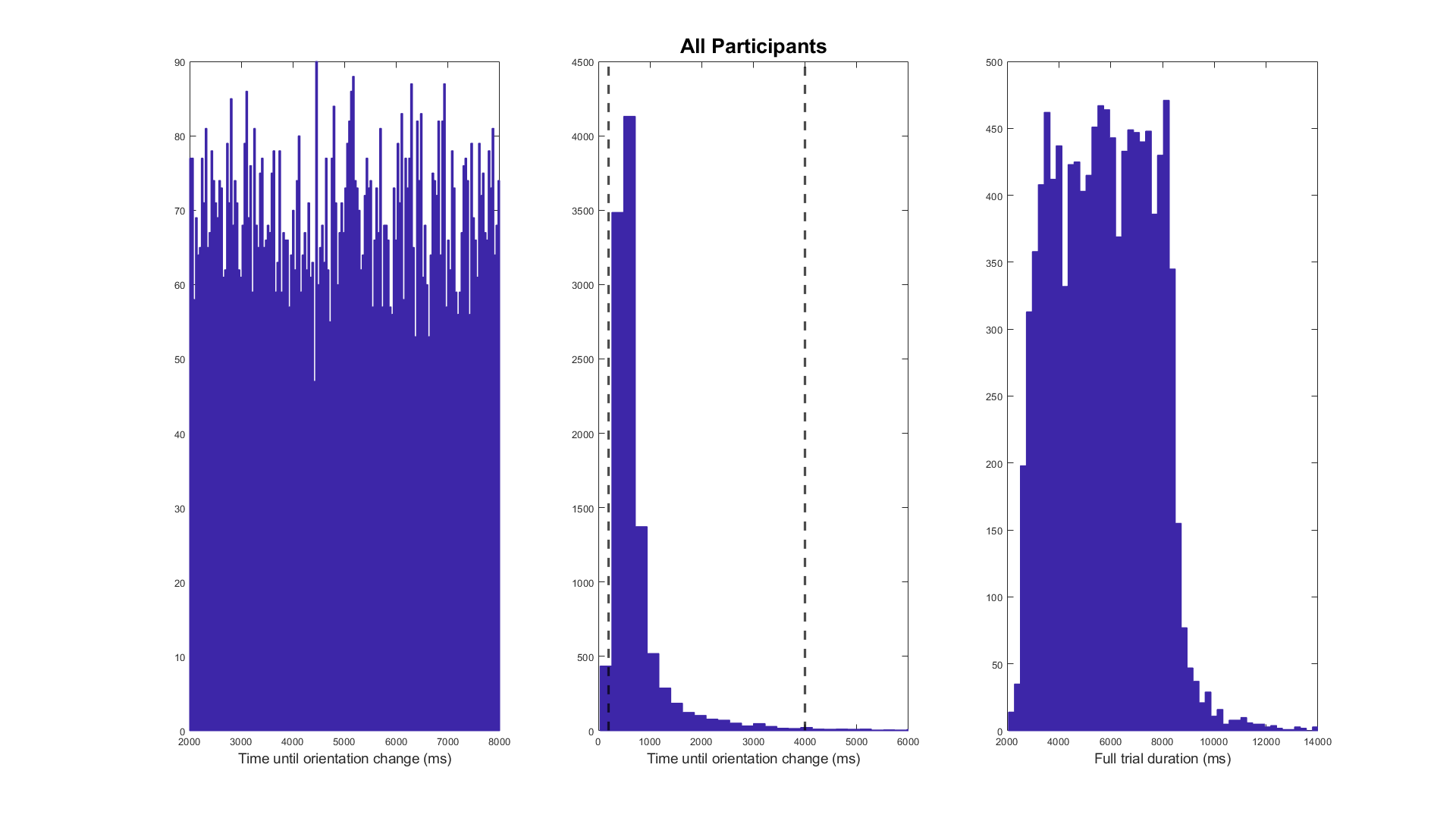
We conducted the same analysis of distributions for Experiment 2. Figure S6 shows the RT-distribution for all participants combined, Figure S7 shows the RT-distribution from the onset of a trial, and Figure S8 shows the RT-distribution from the orientation change for each participant in Experiment 2. Figure S8 clearly shows that Participant 18 repeatedly hit the mouse button supporting our exclusion of that participant’s data. In line with Experiment 1, the distributions are predominantly ex-Gaussian but we ran the same analysis outlined above to check whether there was justification for removing any other participants.

*Figure S6.* Response time distributions for all participants in Experiment 2. Vertical dotted lines (middle panel) show the cut-offs used in our experiment (200 ms – 4000 ms).


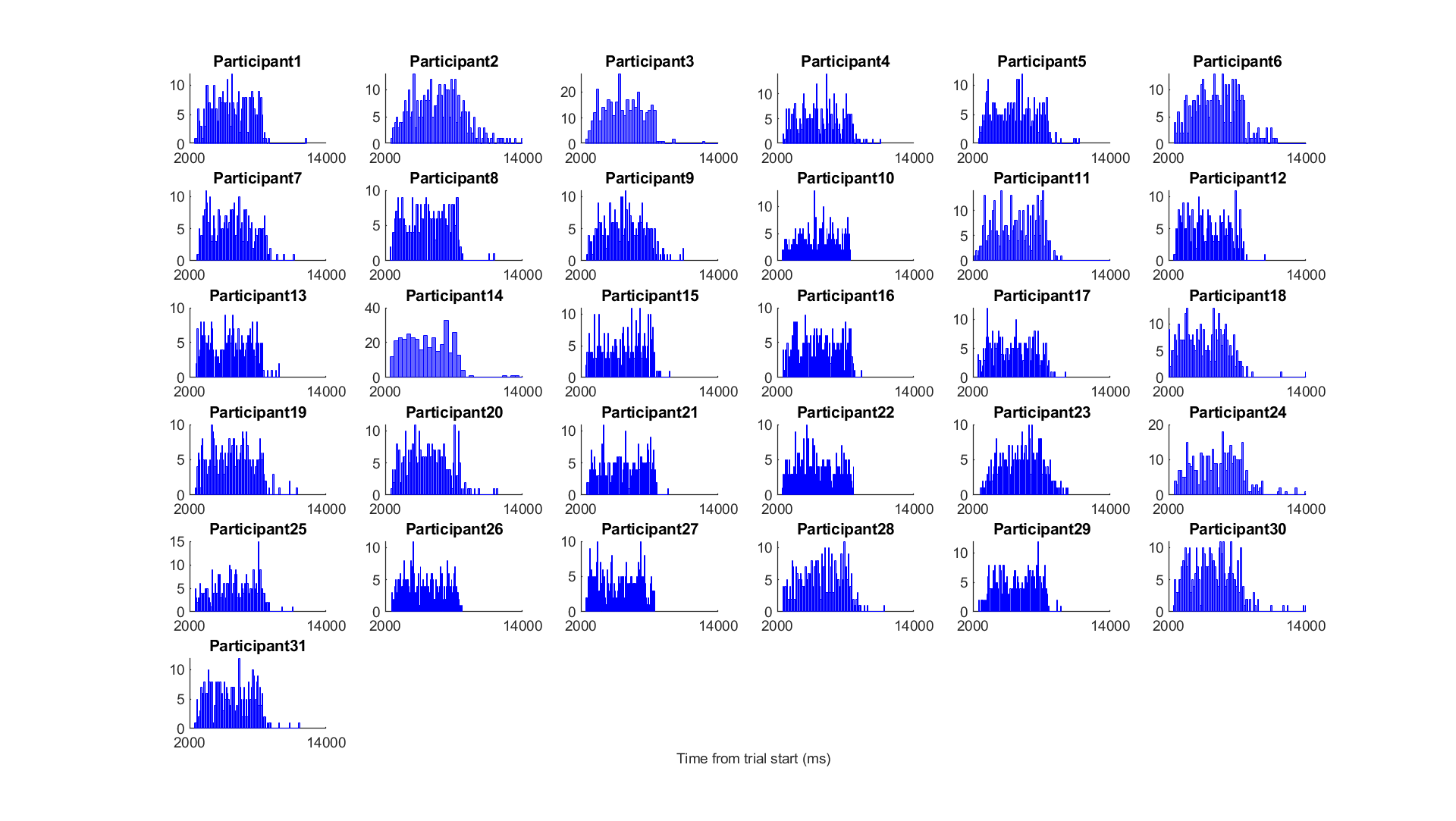


Figure S7*.* Response time distributions from trial start to participant response for each individual participant in Experiment 2.


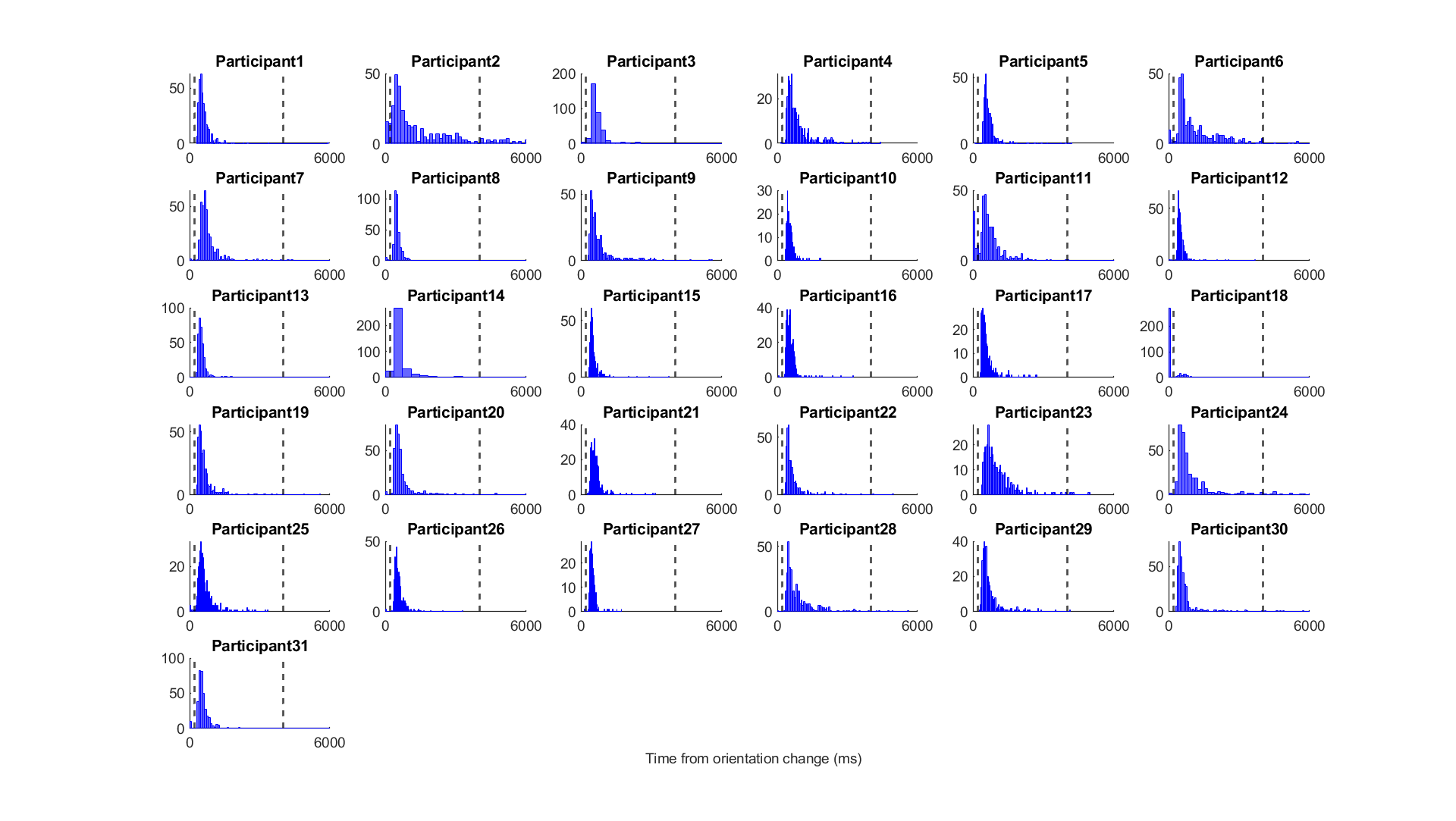
*Figure S8.* Response time distributions from orientation change to participant response for each individual participant in Experiment 2. Vertical dotted lines represent the cut-offs used in our experiment (200 ms – 4000 ms).

Figure S9 shows the cumulative frequency of the vincentized data for each individual participant. The vast majority of participants show a linear slope in the cumulative speed of their response. Upon a further inspection, we removed Participants 11 and 18 from the analysis reported in the manuscript. In line with Experiment 1, we take the median RT at the participant level to avoid any particularly long RT trials exerting a large influence.


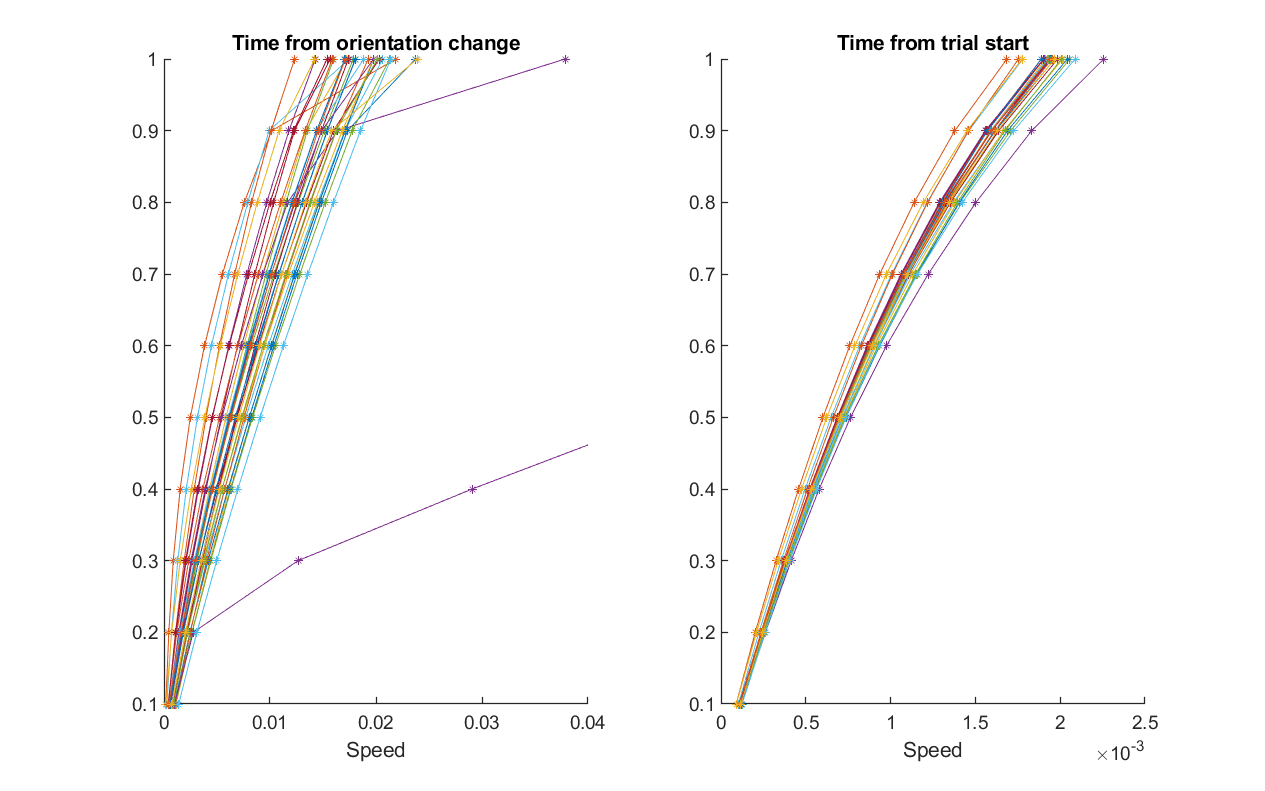


*Figure S9.* Vincentized data for each participant in Experiment 1. Left panel shows RT from orientation change; right panel shows RT from trial start.
